# A novel role of follicle-stimulating hormone (FSH) in various regeneration-related functions of endometrial stem cells

**DOI:** 10.1038/s12276-022-00858-1

**Published:** 2022-09-18

**Authors:** Se-Ra Park, Soo-Rim Kim, Seong-Kwan Kim, Jeong-Ran Park, In-Sun Hong

**Affiliations:** 1 Department of Health Sciences and Technology, GAIHST, Gachon University, Incheon, 21999 Republic of Korea; 2 Department of Molecular Medicine, School of Medicine, Gachon University, Incheon, 406-840 Republic of Korea; 3 Division of Science Education, Kangwon National University, Chuncheon-si, 24341 Republic of Korea

**Keywords:** Adult stem cells, Reproductive disorders

## Abstract

Follicle-stimulating hormone (FSH) promotes the production and secretion of estrogen, which in turn stimulates the growth and maturation of ovarian follicles. Therefore, consecutive FSH treatment to induce ovarian hyperstimulation (superovulation) is still considered the most cost-effective option for the majority of assisted reproductive technologies (ARTs). However, a relatively high cancellation rate and subsequent low pregnancy outcomes (approximately 15%) are the most challenging aspects of this FSH-based ART. Currently, the main cause for this low implantation rate of FSH-based ART has not yet been revealed. Therefore, we hypothesized that these high cancellation rates with FSH-based superovulation protocols might be associated with the harmful effects of consecutive FSH treatment. Importantly, several recent studies have revealed that tissue-resident stem cell deficiency can significantly reduce cyclic endometrial regeneration and subsequently decrease the pregnancy outcome. In this context, we investigated whether FSH treatment could directly inhibit endometrial stem cell functions and consequently suppress endometrial regeneration. Consistent with our hypothesis, our results revealed for the first time that FSH could inhibit various regeneration-associated functions of endometrial stem cells, such as self-renewal, migration, and multilineage differentiation capacities, via the PI3K/Akt and ERK1/2 signaling pathways both in vitro and in vivo.

## Assisted reproduction: A downside to treatment with follicle-stimulating hormone

Follicle-stimulating hormone (FSH) is commonly administered to treat female infertility by stimulating the ovaries, but FSH treatment can also inhibit key cellular and physiological processes required for successful pregnancy. In the light of pregnancy outcomes as low as 15 percent after FSH-based assisted reproduction technologies, In-Sun Hong at Gachon University, Incheon, South Korea, and colleagues investigated the effects of FSH. Working with cultured human stem cells from the lining of the uterus, they found that FSH could inhibit multiple cellular regenerative functions that normally maintain this lining. They also identified a specific molecular signaling pathway involved in mediating these inhibitory effects. Studies in mice supported the cell culture results. The findings could help improve infertility treatment strategies by guiding research into methods to alleviate the unwanted effects of FSH.

## Introduction

FSH is a neuroendocrine hormone secreted from the anterior pituitary that plays an essential role in female reproduction [1]. This heterodimeric glycoprotein is produced from neurons within the anterior pituitary and secreted to affect ovarian follicles. In turn, FSH acts on follicular granulosa cells to induce the production and secretion of the key steroid hormone estrogen, which stimulates the growth and maturation of ovarian follicles and subsequently improves the quality and recovery rate of oocytes [2, 3]. Therefore, at the level of the pituitary-ovarian axis, consecutive FSH treatment to induce hyperovulation is widely used as the gold standard protocol for most assisted reproductive technologies (ARTs) [4, 5]. However, embryo implantation and successful pregnancy rates in patients with infertility receiving prolonged FSH treatment are only 5% and 15%, respectively [6]. Currently, the main causes for these low pregnancy rates with prolonged FSH-based ART have not been revealed.

Successful embryo implantation and subsequent pregnancy outcomes essentially require a receptive uterine endometrium [7]. Poor endometrial receptivity (thickness < 7 mm) is a major risk factor for implantation failure in patients who experience repeated rounds of unsuccessful ART cycles or two or more miscarriages [8, 9]. The endometrium, an inner lining of the cavity of the uterus, is one of the most dramatically regenerating tissues. It undergoes rapid cyclic changes up to 9–11 mm within the proliferative phase (typically on Days 5–13) during the menstrual cycle [10]. Similar to other rapidly growing replacement tissues, resident stem cells play an essential role in the dynamic reconstruction of the uterine endometrium [11, 12]. Consistently activated and recruited tissue-resident stem cells that can give rise to various types of endometrial cells are essential to achieve endometrial regeneration and subsequent successful pregnancy [13]. Lucas et al. have previously found that a deficiency in actively self‐renewing endometrial stem cell subpopulations can markedly limit the regenerative capacity of the uterine endometrium and subsequently increase the risk of premature birth or miscarriage [13]. Interestingly, the functional FSH receptor (FSHR) is highly expressed in the endometrial lining throughout the menstrual cycle [14–16]. Enhanced expression of FSHR in the human endometrium can provide new insight into the possible direct effects of FSH on endometrial regeneration, which is mainly maintained by tissue-resident endometrial stem cells. Therefore, we hypothesized that in addition to its previously known functions in stimulating ovarian growth and subsequent hyperovulation through the pituitary-ovarian axis, prolonged FSH treatment could directly damage tissue-resident endometrial stem cells, which in turn would decrease endometrial receptivity during FSH-based hyperovulation. Currently, the direct effects of FSH on various endometrial stem cell functions and the underlying molecular mechanisms remain unknown.

## Materials and methods

### Isolation and culture of human endometrial stem cells from endometrial tissues

Human endometrial stem cells were obtained from endometrial tissues of uterine fibroid patients with written informed consent from the patients and approval of the Gachon University Institutional Review Board (IRB No: GAIRB2018-134). Endometrial tissue was minced into small pieces, and then the small pieces were digested in DMEM containing 10% FBS and 250 U/ml type I collagenase for 5 h at 37 °C in a rotating shaker. The digestion mixture was then filtered through a 40-µm cell strainer to separate stromal-like stem cells from epithelial gland fragments and undigested tissue. Isolated endometrial stem cells were then cultured following previously established protocols [17]. The endometrial cells were cultured in StemPro® MSC SFM CTS™ (GIBCO, Cat No.: A1033201) at 37 °C under 5% CO_2_ in air. The culture medium was changed every 2 or 3 days.

### Cell proliferation assay

The MTT assay was used to determine the growth-inhibiting capacity of FSH (Cloud clone Corp., Cat. No.: RPD017Hu01), according to the manufacturer’s protocol (Sigma, Cat. No.: M5655). Cells (1 × 10^4^ cells/well) were seeded in 96-well plates. After 24 h of incubation, the cells were treated with FSH or vehicle for 72 h. The viable cells were measured at 570 nm using a Versa Max microplate reader.

### In vitro cell migration assay

The inhibitory effects of FSH on the migration capacity of endometrial stem cells were analyzed by measuring the number of cells that migrated in response to FSH treatment divided by the number of spontaneously migrating cells. Cells were plated at 1 × 10^5^ cells/well in 200 μL of culture medium in the upper chambers of permeable Transwell supports (Corning Inc., Corning, NY, USA) to track the migration of cells. The Transwell chambers had 8.0-μm pores in 6.5-mm-diameter polycarbonate membranes and used a 24-well plate format. Noninvasive cells on the upper surface of each membrane were removed by scrubbing with laboratory paper. Migrated cells on the lower surface of each membrane were fixed with 4% paraformaldehyde for 5 min and stained with hematoxylin for 15 min. Later, the number of migrated cells was counted in three randomly selected fields of each well under a light microscope at 50X magnification. The difference in each group is shown as the fold change.

### Protein isolation and western blot analysis

Protein expression levels were determined by western blot analysis as previously described [18]. Cells were lysed in a buffer containing 50 mM Tris, 5 mM EDTA, 150 mM NaCl, 1 mM DTT, 0.01% NP 40, and 0.2 mM PMSF. The protein concentrations of the total cell lysates were measured by using bovine serum albumin as a standard. Samples containing equal amounts of protein were separated via sodium dodecyl sulfate‒polyacrylamide gel electrophoresis (SDS-PAGE) and then transferred onto nitrocellulose membranes (Bio-Rad Laboratories). The membranes were blocked with 5% skim milk in Tris-buffered saline containing Tween-20 at RT. Then, the membranes were incubated with primary antibodies against MMP-2 (Cell Signaling #4022), MMP-9 (Cell Signaling #13667), total PI3K (Cell Signaling #4292), phospho-PI3K (Cell Signaling #4228), total Akt (Cell Signaling #4491), phospho-Akt (Cell Signaling #4060), total-ERK1/2 (Cell Signaling #9012), phospho-ERK1/2 (Cell Signaling #9101), total FAK (Santa Cruz, sc-558), phospho-FAK (Santa Cruz, sc-11765), or β-actin (Abcam, ab189073) overnight at 4 °C and then with HRP-conjugated goat anti-rabbit IgG (BD Pharmingen, 554021) or goat anti-mouse IgG (BD Pharmingen, 554002) secondary antibodies for 60 min at RT. Antibody-bound proteins were detected using enhanced chemiluminescence (ECL) reagents.

### Adipogenic differentiation

Endometrial stem cells were incubated in DMEM low-glucose medium supplemented with 500 µM methylxanthine, 5 µg/mL insulin, and 10% FBS. Endometrial stem cells were grown for 3 weeks, with medium replacement twice a week with or without FSH treatment. Lipid droplet formation was confirmed by oil red O staining. Relative quantification of lipid droplet formation was determined by absorbance measurement at 500 nm.

### Osteogenic differentiation

Endometrial stem cells were incubated in DMEM high-glucose medium supplemented with 0.1 µM dexamethasone, 10 mM β-glycerophosphate, 50 µM ascorbate and 10% FBS. Endometrial stem cells were grown for 3 weeks, with medium replacement twice a week with or without FSH treatment. Differentiated cells were stained with Alizarin Red S to detect *de novo* formation of bone matrix. Alizarin red S in samples was quantified by measuring the optical density (OD) of the solution at 570 nm.

### Real-time PCR

Total RNA from endometrial stem cells was extracted using TRIzol reagent (Invitrogen) according to the manufacturer’s protocol. Real-time PCR was performed using a Rotor-Gene Q (Qiagen). The reaction was subjected to amplification cycles of 95 °C for 20 s, 60 °C for 20 s and 72 °C for 25 s. The relative mRNA expression of the selected genes was normalized to that of PPIA and quantified using the ΔΔCT method. The sequences of the PCR primers are listed in Table 1.

**Table 1:** Primer sequences for quantitative RT‒PCR.

Gene	GenBank No.	Direction	Primer sequence
Human PPIA	NM_021130	F	TGCCATCGCCAAGGAGTAG
		R	TGCACAGACGGTCACTCAAA
Human NANOG	NM_024865	F	TGGGATTTACAGGCGTGAGC
		R	AAGCAAAGCCTCCCAATCCC
Human OCT4	NM_002701	F	AGCCCTCATTTCACCAGGCC
		R	TGGGACTCCTCCGGGTTTTG
Human SOX2	NM_003106	F	AAATGGGAGGGGTGCAAAAGAGGAG
		R	CAGCTGTCATTTGCTGTGGGTGATG
Human FSHR	NM_000145	F	ATGGAAGCCAGCCTCACCTAT
		R	TCTGACCCCTAGCCTGAGTCATA
Mouse NANOG	NM_028016	F	GCCTTACGTACAGTTGCAGC
		R	TCACCTGGTGGAGTCACAGA
Mouse OCT4	NM_013633	F	GCATTCAAACTGAGGCACCA
		R	AGCTTCTTTCCCCATCCCA
Mouse SOX2	NM_011443	F	GAAGCGTGTACTTATCCTTCTTCAT
		R	GAGTGGAAACTTTTGTCCGAGA

### FSH receptor (FSHR) knockdown

Small hairpin RNA targeting FSHR (shRNA: accession No. NM_000145) and scrambled shRNA (shCTRL) were purchased from Bioneer (Daejeon, South Korea). For efficient shRNA transfection, reverse transfection was performed using Lipofectamine 2000 (Invitrogen, Cat No: 52887) according to the manufacturer’s protocol. We chose the FSHR shRNA that was most effective at the mRNA level from five shRNAs designed from the target sequence based on qRT-PCR analysis.

### Ingenuity pathway analysis (IPA)

FSH-, Akt1-, MAPK1/3 (ERK1/3)-, IGF1R-, PDGFA-, PDGFRB-, EGF-, EGFR-, or KIT-related gene analyses were performed with IPA version 2.0 software (Ingenuity Systems, Redwood City, CA). Differentially expressed genes (*t* test, *P* < 0.005) between nonproliferative cells and proliferative cells were subjected to FSH (GSE50831, GSE36133)-, Akt1 (GSE62564)-, MAPK1/3 (GSE21034, GSE 44752)-, IGF1R (GSE63074)-, PDGFA (GSE28878)-, PDGFRB (GSE36133)-, EGF (GSE62564)-, EGFR (GSE62564)-, or KIT (GSE62564)-related gene analysis. The significance of each factor was measured by Fisher’s exact test (*p* value), which was used to identify differentially expressed genes from the microarray data that overlapped with genes known to be regulated by a factor. The activation score (Z score) was used to show the status of predicted factors by comparing the observed differential regulation of genes (“up” or “down”) in the microarray data relative to the literature-derived regulation direction, which can be either activating or inhibiting.

### Analysis of the GEO database

GEO (https://www.ncbi.nlm.nih.gov/geo/) is a freely distributed database repository of high-throughput gene expression data generated by genome hybridization arrays, chip sequencing and DNA microarrays [19, 20]. Researchers provide their experimental results in four categories: experimental designs, samples, platforms, and raw data. Clinical or experimental samples within each dataset are further organized based on various experimental subgroups, such as treatment, physiologic condition, and disease state. These categorized biological data are presented as “GEO profiles”, which include the dataset title, the gene annotation, a chart depicting the expression levels, and the rank for that gene across each sample [21]. Gene expression data were selected from GEO datasets according to multiple parameters, such as tissues, cancers, diseases, genetic modifications, external stimuli, or development. The expression profiles of FSH receptor (FSHR), Akt, MAPK1 (ERK1), MAPK3 (ERK3), IGF1R, PDGFA, PDGFRB, EGF, EGFR, and KIT under various physiological conditions were analyzed according to previously established procedures [21].

### Evaluation of the effects of FSH treatment in an animal model

All of the animal experiments were approved and conducted in accordance with the Institutional Animal Care and Use Committee (IACUC) (LCDI-2019-0012) of Gachon University. The mice were randomly divided into the control and FSH treatment (100 IU/mouse for 7 consecutive days intravenously) groups. The mice were anesthetized and exsanguinated by cardiac puncture, and then stem cells were isolated from uterine and adipose tissues. The isolation of mouse uterine tissue- or adipose tissue-derived stem cells was approved and conducted in accordance with the Institutional Animal Care and Use Committee (IACUC) (LCDI-2019–0012) of the Lee Gil Ya Cancer and Diabetes Institute of Gachon University. Uterine or adipose tissue was minced into small pieces, and then the small pieces were digested in DMEM containing 10% FBS and 250 U/ml type I collagenase for 5 h at 37 °C. The digestion mixture was then filtered through a 40-µm cell strainer. The endometrial cells were cultured in StemPro® MSC SFM CTS™ (GIBCO, Cat No.: A1033201) at 37 °C under 5% CO_2_ in air. The culture medium was changed every 2 or 3 days. For further experiments, isolated stem cells from the endometrium were cultured and expanded in vitro with continuous exposure to FSH (30 IU/ml) to properly mimic the physiological conditions of FSH exposure in vivo.

### Statistical analysis

All in vivo and in vitro data are presented as the mean ± S.D. of three independent experimental repeats. All statistical data were analyzed with GraphPad Prism 5.0 (GraphPad Software, San Diego, CA) and evaluated using two-tailed Student’s *t* tests. Values of *P* < 0.05 were considered to indicate statistical significance. The variance between the groups was not significant. None of the samples were excluded.

## Results

### FSH significantly inhibits various regenerative potential-associated functions of endometrial stem cells in vitro

**Fig. 1:**
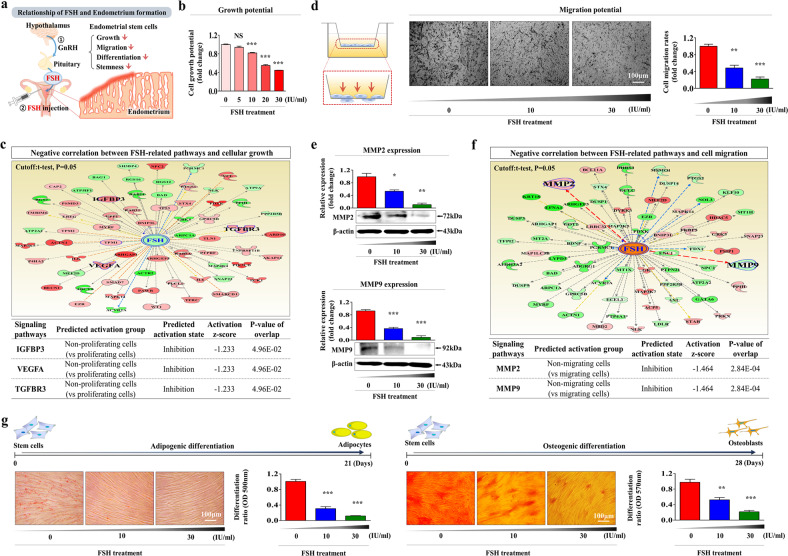
FSH treatment markedly reduces the growth, migratory, and multilineage differentiation potential of endometrial stem cells in vitro. We hypothesized that FSH treatment would suppress various tissue repair and regeneration capacities of endometrial stem cells, such as the self-renewal, migration, and multilineage differentiation capacities (**a**). The inhibition of self-renewal capacity by treatment with FSH at different doses (5 IU, 10 IU, 20 IU, and 30 IU) was analyzed at 72 h after treatment by MTT assays. The cell proliferation rate (%) was analyzed as the viability of FSH-treated cells as a percentage of control cells treated with vehicle (**b**). The activation status (whether intermediate, inactivate, or activate) of various FSH (GSE50831)-related genes in proliferative and nonproliferative cells was assessed using the ingenuity pathway analysis (IPA) system (**c**). Endometrial stem cells were treated with FSH at 10 IU or 30 IU for 72 h. The inhibitory effects of FSH exposure on migration ability were then analyzed using transwell assays. FSH exposure markedly reduced cell migration across the membrane of transwells (**d**). Protein levels of positive regulatory factors of cell migration (MMP-2/9) in response to FSH exposure were analyzed by western blotting (**e**). The activation status (intermediate, inactivate, or activate) of various FSH (GSE36133)-related genes in migrating and nonmigrating cells was assessed using the IPA system (**f**). Cells were cultured for 14 days in adipocyte or osteoblast differentiation medium with or without FSH (10 IU or 30 IU) treatment. The inhibitory effects of FSH treatment on the adipocyte and osteoblast differentiation of endometrial stem cells were analyzed by oil red O staining and alizarin red S staining, respectively. Relative quantification of calcium deposition and lipid droplet (LD) secretion from differentiating cells was performed by measuring the absorbance of solubilized cells at 500 nm and 570 nm, respectively (**g**). β-Actin was used as an internal control. All experiments were performed in triplicate. Data are presented as the mean ± standard deviation (SD). *p < 0.05; **p < 0.005; and ***p < 0.001 (two-sample *t* test).

Human endometrial stem cells were isolated from hysterectomy samples and properly cultured as described in our previous studies [22–26] (Supplementary Fig. 1a). The pluripotency of isolated cells was assessed by flow cytometry using various negative (CD44 and CD45) and positive (CD34, CD73, CD105, CD140b, CD146, and susD2) cell surface markers (Supplementary Fig. 1b). Additionally, their multilineage differentiation capacity into other types of cells was analyzed by inducing adipocyte (Supplementary Fig. 1c) and osteoblast (Supplementary Fig. 1d) differentiation. A schematic summary showing the inhibitory effects of FSH on endometrial stem cells is described in Fig. 1a. We investigated whether FSH could restrict the regenerative capacity of the endometrium by suppressing several critical functions of endometrial stem cells. The approximate IC_50_ value of FSH was determined using a dose‒response curve. In human endometrial stem cells, the IC_50_ value was approximately 30 IU (Supplementary Fig. 2). The results showed that FSH significantly reduced the self-renewal capacity of endometrial stem cells in a dose-dependent manner (Fig. 1b). To confirm whether FSH-related signaling integrity was associated with self-renewal capacity, we analyzed the signaling networks associated with the activities of multiple genes using ingenuity pathway analysis (IPA). Several positive regulators of FSH (IGFBP3, VEGFA, and TGFBR3) known to be associated with cell proliferation and survival were inhibited in proliferating cells (Fig. 1c). FSH treatment also significantly suppressed the migration potential of endometrial stem cells in a dose-dependent manner (Fig. 1d). To further investigate the suppressive effect of FSH on the migration potential of endometrial stem cells, the expression levels of MMP-2 and MMP-9, which are known to promote cell invasion and migration, were assessed using western blotting (Fig. 1e). We further investigated the effects of FSH on the activity of MMP-2 and MMP-9 via gelatin zymography. Consistently, the activities of MMP-2 and MMP-9 were significantly decreased in the FSH treatment group compared with the nontreated control group (Supplementary Fig. 3). IPA also revealed negative correlations between FSH-related signaling integrity and MMP-2/9 activities (Fig. 1f). In addition, FSH treatment markedly reduced the transdifferentiation potential of endometrial stem cells into adipocytes and osteoblasts in vitro (Fig. 1g). The expression levels of the pluripotency-related genes NANOG, OCT4, and SOX2 were also significantly decreased by FSH treatment (Supplementary Fig. 4). These results indicate that FSH can possibly restrict the regenerative capacity of the endometrium by inhibiting various beneficial functions of endometrial stem cells, such as the proliferation, migration potential, pluripotency, and multilineage differentiation capability.

### The suppressive effects of FSH on various regenerative potential-associated functions of endometrial stem cells are mediated by its cognate receptor FSHR

**Fig. 2:**
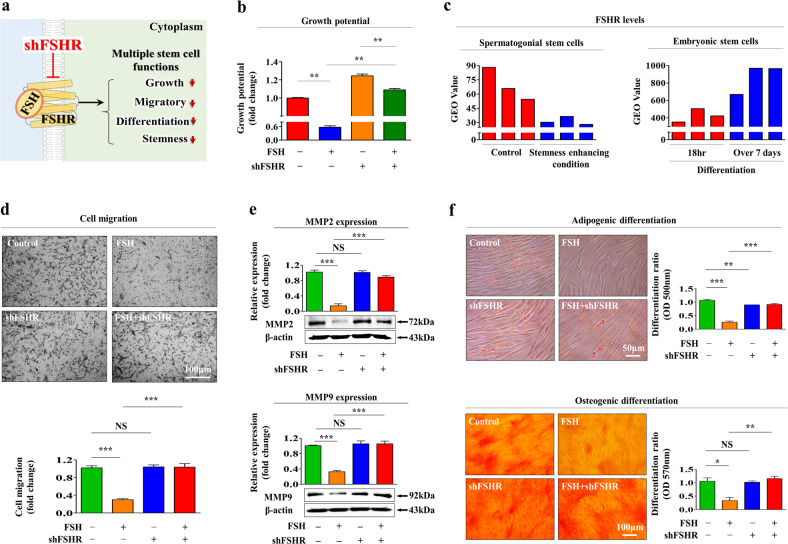
FSH exerts its inhibitory effects on various endometrial stem cell functions through its cognate receptor FSHR. A schematic summary of the role of FSH receptor (FSHR) in regulating the FSH-induced inhibitory effects on endometrial stem cells is shown (**a**). Endometrial stem cells were treated with 30 IU/ml FSH alone or were concomitantly transfected with an shRNA specifically targeting FSHR. The subsequent decrease in cell growth was analyzed with MTT assays (**b**). The GEO database (https://www.ncbi. nlm.nih.gov/geo/) was used to assess the relationship between FSHR levels and several conditions of stem cells (**c**). The inhibitory effects of FSHR knockdown on FSH-mediated changes in migration ability were also measured by Transwell assays (**d**) and western blotting for MMP-2 and MMP-9 (**e**). The abolishing effects of FSHR depletion on the roles of FSH in inhibiting adipocyte and osteoblast differentiation were assessed by oil red O staining and alizarin red S staining, respectively (**f**). β-Actin was used as an internal control. All experiments were performed in triplicate. Data are presented as the mean ± standard deviation (SD). *p < 0.05; **p < 0.005; and ***p < 0.001 (two-sample *t* test).

The effects of FSH are known to be mediated by its cognate receptor FSHR, which belongs to the superfamily of G-protein coupled receptors (GPCRs) in other cell types [27]. To assess whether FSH could exert its functions through its cognate receptor in endometrial stem cells, FSHR was stably depleted by transfection with a specific shRNA targeting FSHR (Supplementary Fig. 5a, b). A schematic summary of the new roles of FSHR as a functional receptor that mediates FSH-induced inhibitory effects on various functions of endometrial stem cells is shown in Fig. 2a. The FSH-induced suppressive effect on self-renewal capacity was significantly reduced by FSHR knockdown (Fig. 2b). We then further investigated the correlations between several physiological conditions of stem cells and FSHR expression levels by analyzing the GEO (Gene Expression Omnibus) repository database. Interestingly, the levels of FSHR were also markedly decreased in a pluripotency-enhancing condition or increased in differentiation-promoting conditions compared with corresponding controls (Fig. 2c). In addition, the FSH-mediated suppressive effects on migration capacities (Fig. 2d) and expression levels of MMP-2 and MMP-9 (Fig. 2e) were markedly abolished by FSHR knockdown. FSHR knockdown also significantly attenuated the FSH-mediated inhibitory effects on transdifferentiation capacities into adipocytes and osteoblasts (Fig. 2f). Consistently, the FSH-mediated effects on the expression levels of pluripotency-related genes such as NANOG, OCT4, and SOX2 were significantly decreased by FSHR depletion (Supplementary Fig. 6a–c). These results suggest that FSHR can mediate the FSH-induced suppressive effects on various tissue repair capacity-related functions of endometrial stem cells.

### The FSH-induced suppressive effects on regeneration capacity-related functions are mediated by the Akt and ERK1/2 signaling pathways

**Fig. 3:**
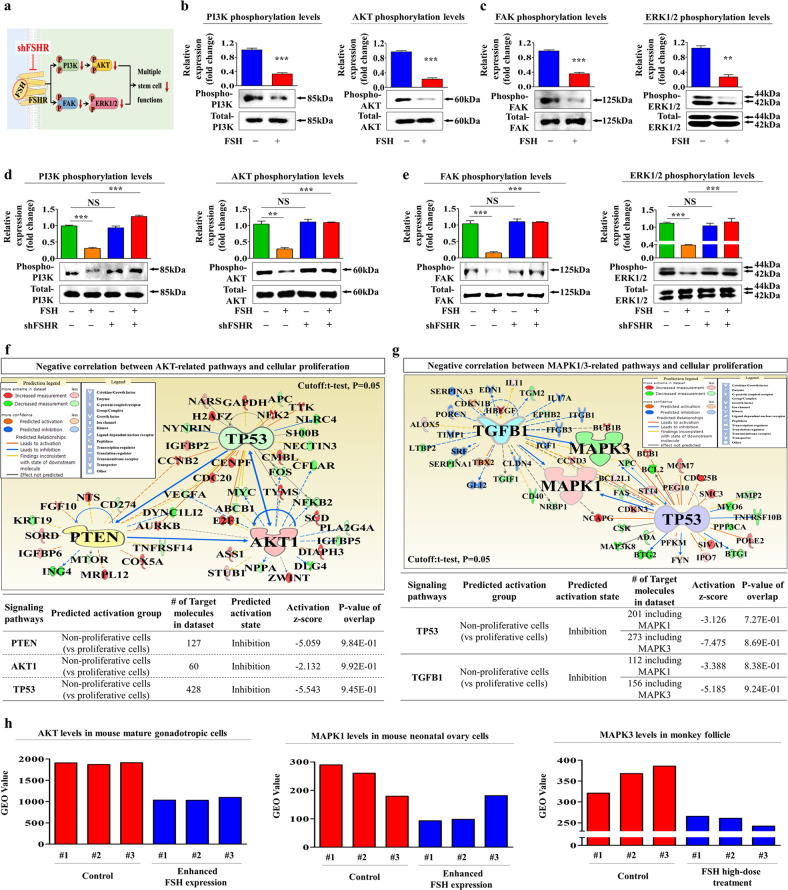
FSH-mediated suppressive effects in endometrial stem cells are regulated through Akt and/or ERK1/2 signaling cascades. A schematic summary of the roles of FAK/ERK1/2 and/or PI3K/Akt signaling cascades in regulating the FSH-mediated inhibitory effects on endometrial stem cells is described (**a**). Cells were treated with or without FSH at 30 IU/ml for 10 min. Treated endometrial stem cells were washed with PBS and then lysed. Subsequent changes in the phosphorylation levels of Akt, PI3K, FAK, and ERK1/2 were measured by western blotting (**b**, **c**). Endometrial stem cells were treated with FSH (30 IU/ml) alone or were concomitantly transfected with an shRNA specifically targeting FSHR. Subsequent changes in the phosphorylation levels of PI3K, Akt FAK, and ERK1/2 were measured by western blotting (**d**, **e**). Differentially activated genes in rapidly proliferating cells and nonproliferating cells were analyzed using IPA software to investigate the activation status (intermediate, inactivate, or activate) of AKT1 (GSE62564) (**f**) or MAPK1/3 (ERK1/3) (GSE21034, GSE44752) (**g**)-associated signaling molecules/transcription factors. Furthermore, the GEO data repository was used to assess the relationship between various FSH-enhancing conditions and the expression levels of AKT or MAPK1/3 (**h**). β-Actin was used as the internal control. All experiments were performed in triplicate. Data are presented as the mean ± standard deviation (SD). *p < 0.05; **p < 0.005; and ***p < 0.001 (two-sample *t* test).

**Fig. 4:**
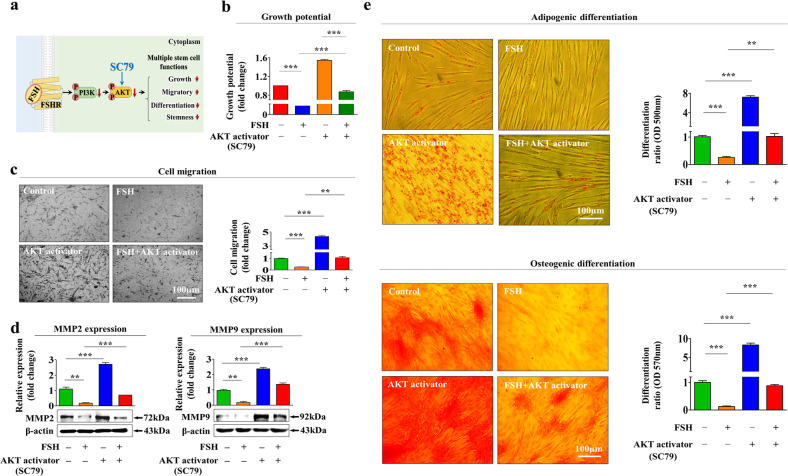
Increased Akt signaling activity abolishes the FSH-induced inhibitory effects on various endometrial stem cell functions. A schematic summary of the role of the PI3K/Akt signaling pathway in mediating the FSH-induced inhibitory effects on endometrial stem cells is described (**a**). Endometrial stem cells were prestimulated with the Akt activator SC79 (10 µM) for 1 h prior to FSH (30 IU/ml) treatment for 48 h. The FSH-induced effects on cell growth were then analyzed by MTT assays (**b**). The attenuating effects of the Akt activator on FSH-induced migration abilities were measured by Transwell assays **(c)** and western blotting for the cell migration regulators MMP-2 and MMP-9 (**d**). Endometrial stem cells were prestimulated with the Akt activator SC79 (10 µM) for 1 h prior to an additional FSH (30 IU/ml) treatment for 48 h. The effects on adipogenic and osteogenic differentiation capacity were then analyzed by oil red O and alizarin red S staining, respectively. Relative quantification of calcium deposition and lipid droplet (LD) secretion from differentiating cells was performed by measuring the absorbance of solubilized cells at 500 nm and 570 nm, respectively (**e**). β-Actin was used as an internal control. All experiments were performed in triplicate. Data are presented as the mean ± standard deviation (SD). *p < 0.05; **p < 0.005; and ***p < 0.001 (two-sample *t* test).

**Fig. 5:**
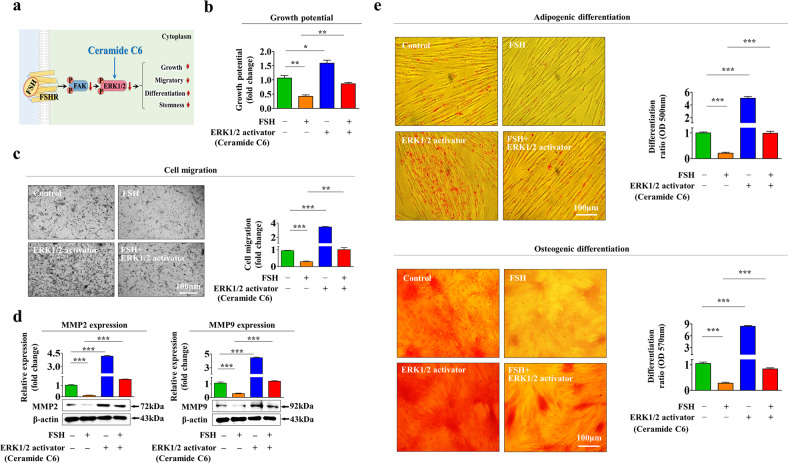
Increased ERK1/2 signaling activity abolishes the FSH-induced inhibitory effects on various endometrial stem cell functions. A schematic summary of the role of the FAK/ERK1/2 signaling pathway in mediating FSH-induced inhibitory effects on endometrial stem cells is described (**a**). Endometrial stem cells were prestimulated with the ERK1/2 activator ceramide C6 (10 µM) for 1 h prior to FSH (30 IU/ml) treatment for 48 h. The FSH-induced effects on cell growth were then analyzed by MTT assays (**b**). The attenuating effects of the ERK1/2 activator C6 (10 µM) on the FSH-induced effects on migration ability were measured by Transwell assays **(c)** and western blotting for the cell migration regulators MMP-2 and MMP-9 (**d**). Endometrial stem cells were prestimulated with the ERK1/2 activator ceramide C6 (10 µM) for 1 h prior to an additional FSH (30 IU/ml) treatment for 48 h. Subsequent effects on the adipogenic and osteogenic differentiation capacity were analyzed by oil red O staining and alizarin red S staining, respectively. Relative quantification of calcium deposition and lipid droplet (LD) secretion from differentiating cells was performed by measuring the absorbance of solubilized cells at 500 nm and 570 nm, respectively (**e**). β-Actin was used as an internal control. All experiments were performed in triplicate. Data are presented as the mean ± standard deviation (SD). *p < 0.05; **p < 0.005; and ***p < 0.001 (two-sample *t* test).

To investigate the molecular mechanisms responsible for the suppressive effects of FSH on the tissue repair capacity-related functions of endometrial stem cells, we analyzed the effects of FSH on the PI3K/Akt and FAK/ERK1/2 signaling pathways known to be associated with the pluripotency/stemness [28], self-renewal ability [29], and migratory capacity [30] of various stem cell types. A schematic diagram showing the roles of the PI3K/Akt and FAK/ERK1/2 signaling pathways in the FSH-induced suppressive effects in endometrial stem cells is shown in Fig. 3a. We assessed whether the PI3K/Akt (Fig. 3b) and FAK/ERK1/2 (Fig. 3c) signaling pathways were inhibited by FSH treatment. We then investigated the effect of FSHR depletion on the FSH-mediated suppression of both signaling pathways. Indeed, FSH-induced inhibitory effects on the PI3K/Akt and FAK/ERK1/2 signaling pathways were significantly abolished by FSHR knockdown (Fig. 3d, e). To further analyze whether these signaling pathways were positively correlated with the self-renewal capacity, we investigated the Akt and ERK (MAPK)1/3-associated signaling networks involved in cell proliferation using IPA. Negative regulators of the Akt signaling pathway, such as TP53 and PTEN, which are known to be associated with a halt in cell division, were markedly reduced in rapidly proliferating cells (Fig. 3f). Negative regulators of the ERK1/3 (MAPK1/3) signaling pathway, such as TP53 and TGFβ1, related to cell growth suppression were also reduced in rapidly proliferating cells (Fig. 3g). In addition, the GEO database repository suggested that the activities of these signaling pathways were clearly suppressed under FSH-enhancing conditions (Fig. 3h). Furthermore, to analyze whether the activation of these signaling molecules attenuated FSH-mediated inhibitory effects on various tissue repair capacity-related functions, we assessed the FSH-induced effects with or without Akt activator SC79 (Fig. 4a) or ERK1/2 activator ceramide C6 (Fig. 5a) treatment in vitro. Interestingly, FSH-induced inhibitory effects on the self-renewal capacity of endometrial stem cells were clearly abolished by SC79 (Fig. 4b) or ceramide C6 (Fig. 5b) prestimulation. Similarly, SC79 (Fig. 4c, d) or ceramide C6 (Fig. 5c, d) prestimulation abolished the FSH-mediated inhibitory effects on the migratory capacity and MMP-2/9 levels. These FSH-induced inhibitory effects on the transdifferentiation capacities into adipocytes and osteoblasts as well as the expression of pluripotency-related genes such as NANOG, OCT4, and SOX2 were also significantly attenuated by SC79 (Fig. 4e and Supplementary Fig. 7a) or ceramide C6 (Fig. 5e and Supplementary Fig. 7b) prestimulation. These results suggest that Akt and ERK1/2 signaling activities might be involved in the FSH-mediated inhibitory effects on various tissue repair capacity-related functions of endometrial stem cells.

### Proteome profiling of FSH-induced multiple growth factors and their interconnected signaling networks

**Fig. 6:**
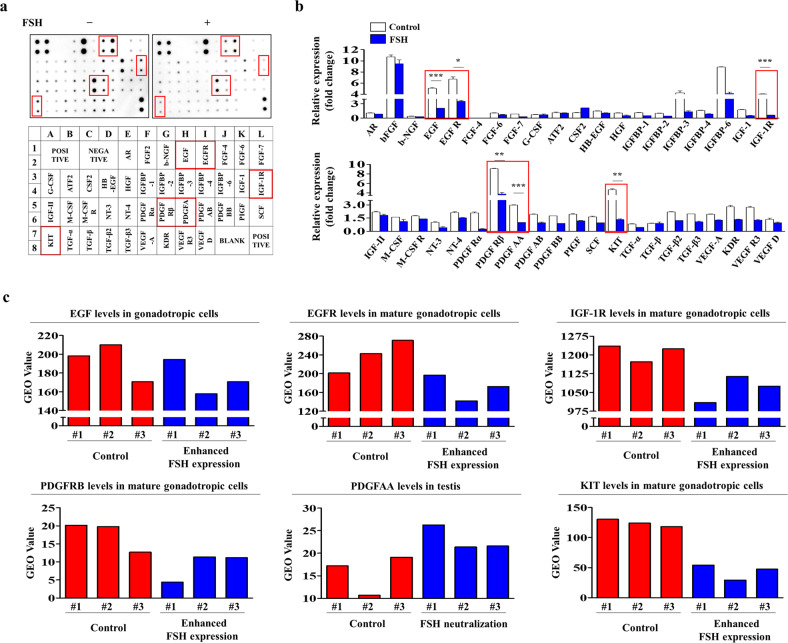
FSH treatment significantly decreases the secretion of various growth factors or cytokines associated with the tissue repair-related signaling network in vitro. A membrane-based human growth factor antibody array was performed using FSH-treated or nontreated medium samples. Nitrocellulose membranes were spotted with 40 different antibodies for multiple cytokines, growth factors, and soluble receptors. Six prominent proteins (EGF, EGFR, IGF-1R, PDGFRβ, PDGF-AA, and KIT) were markedly decreased in medium samples from FSH-treated endometrial stem cells (**a**, **b**). Additionally, the GEO data repository was analyzed to assess correlations between the expression levels of six prominent proteins and FSH treatment (**c**). All experiments were performed in triplicate. Data are presented as the mean ± standard deviation (SD). *p < 0.05; **p < 0.005; and ***p < 0.001 (two-sample *t* test).

To analyze major secreted growth factors associated with the suppressive effects of FSH on endometrial stem cells, we assessed FSH-mediated secretion of various proteins using multiplex antibody arrays to detect cytokines, chemokines, and growth factors. We detected changes in 40 different secreted proteins from FSH-treated endometrial stem cells and nontreated stem cells. Secreted levels of six prominent proteins [epidermal growth factor (EGF), epidermal growth factor receptor (EGFR), insulin-like growth factor 1 receptor (IGF-1R), platelet-derived growth factor receptor-β (PDGFRβ), platelet-derived growth factor AA (PDGF-AA), and tyrosine-protein kinase (KIT, CD117)] associated with Akt and ERK1/2 signaling pathways were substantially reduced by FSH treatment, whereas only minor changes were observed for other secreted proteins (Fig. 6a, b). These results suggest that these secreted proteins, at least partially, are involved in the FSH-mediated inhibitory effect on the Akt and ERK1/2 signaling pathways and its subsequent suppression of various tissue repair capacity-related functions. The GEO dataset also revealed that the expression levels of these six prominent secreted proteins were reduced under various FSH-enhancing conditions (Fig. 6c). We further investigated the activation status of six prominent factors and their related signaling networks regulating self-renewal ability and the cell cycle using IPA software. Positive regulators of IGF-1R, such as Akt and ERK1/2, were highly activated in proliferative cells (Supplementary Fig. 8). Positive regulators of PDGFA, such as EGR1, MAP2K1/2 and IL1A, were also largely activated in proliferative cells (Supplementary Fig. 9). Positive regulators of PDGFRB, such as MYC and BRD4, were activated in proliferative cells (Supplementary Fig. 10). Negative regulators of EGF, such as PTEN and TGFB1, were suppressed in proliferative cells (Supplementary Fig. 11). Positive regulators of EGFR, such as YAP1 and NF-kB, were activated in proliferative cells (Supplementary Fig. 12). Negative regulators of KIT, such as RB1 and TNF, were activated in proliferative cells (Supplementary Fig. 13). These results indicate that these six prominent secreted proteins might act as potent upstream activators of the Akt and ERK1/2 signaling pathways to mediate the inhibitory effects of FSH.

### FSH suppresses various tissue repair-related functions of endometrial stem cells in vivo and subsequent regeneration of injured endometrial tissue

**Fig. 7:**
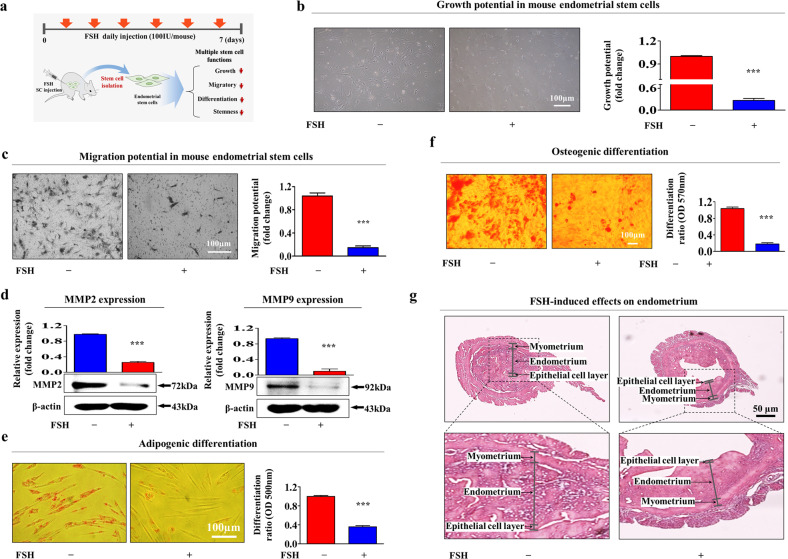
FSH treatment significantly suppresses various tissue repair capacities of endometrial stem cells in vivo. A schematic diagram of the overall experimental protocols as described in the ‘Materials and Methods’ section is presented (**a**). Mice were intravenously treated with FSH (100 IU/mouse daily for 7 consecutive days). Endometrial stem cells were then isolated from endometrial tissues using our collagenase-based primary culture method. After isolation, mouse endometrial stem cells were cultured in vitro either under continuous FSH (30 IU/ml) treatment or non-FSH treatment conditions to properly mimic the in vivo environment of FSH exposure. Subsequent inhibition of cell proliferation was assessed by MTT assays (**b**). FSH-mediated suppression of migration capacity in vivo was then measured using Transwell assays (**c**) and western blotting for MMP-2 and MMP-9 (**d**). FSH-mediated suppression effects on adipocyte (**e**) and osteoblast (**f**) differentiation in vivo were assessed by oil red O staining and alizarin red S staining, respectively. The relative quantification of calcium deposition and lipid droplet (LD) secretion from differencing cells was performed by measuring the absorbance of solubilized cells at 500 nm and 570 nm, respectively. Uterine endometrial tissue samples from FSH-treated or nontreated mice were collected and then fixed in 10% buffered formalin for 48 h. Paraffin sections were then stained with hematoxylin and eosin (H&E) solution. Histological evaluation showed that the functional layer of endometrial tissues was markedly reduced by consecutive FSH exposure in vivo (**g**). β-Actin was used as an internal control to normalize protein expression. All experiments were performed in triplicate. Data are presented as the mean ± standard deviation (SD). *p < 0.05; **p < 0.005; and ***p < 0.001 (two-sample *t* test).

Our in vitro results indicated that consecutive administration of FSH to induce superovulation during IVF therapy might inhibit tissue repair-related functions of resident stem cells. Therefore, we analyzed whether FSH could suppress various tissue repair-related functions of endometrial stem cells in vivo, thus subsequently reducing the regeneration of injured endometrial tissue in an animal model. To mimic FSH-based superovulation protocols during the IVF process, we intraperitoneally administered FSH (100 IU/mouse) to mice for seven consecutive days (seven times). Tissue-resident stem cells were then isolated from the endometrium (Fig. 7a). Consistently, our in vivo results suggested that consecutive FSH treatment remarkably inhibited the growth potential of tissue-resident endometrial stem cells (Fig. 7b). Additionally, the transwell assay and western blotting results revealed the inhibitory effect of FSH on the migration capacity of endometrial stem cells (Fig. 7c) and the expression levels of MMP-2 and MMP-9 (Fig. 7d) in vivo. Furthermore, FSH significantly suppressed their ability to differentiate into adipocytes (Fig. 7e) and osteoblasts (Fig. 7f) in vivo. Exogenous FSH exposure also significantly decreased the expression levels of pluripotency-associated genes such as NANOG, OCT4, and SOX2 in vivo (Supplementary Fig. 14a–c). Importantly, we further assessed whether consecutive FSH exposure could affect the tissue repair capacity of the endometrium known to be primarily maintained by tissue-resident stem cells. Histological analysis of the endometrium revealed that the thickness of its functional layer was significantly decreased by consecutive FSH exposure (Fig. 7g). Furthermore, we analyzed whether FSH exposure also inhibited various tissue repair capacity-related functions of other tissue-resident stem cells, such as adipose tissue-derived stem cells (Supplementary Fig. 15a). Consistently, FSH exposure significantly inhibited the self-renewal (Supplementary Fig. 15b), migration (Supplementary Fig. 15c, d), and multilineage differentiation potential (Supplementary Fig. 15e, f) of adipose tissue-derived stem cells. Additionally, the expression levels of pluripotency-associated genes such as NANOG, OCT4, and SOX2 were significantly decreased by FSH exposure in adipose tissue-derived stem cells in vivo (Supplementary Fig. 15g). Taken together, these results indicate that consecutive FSH exposure during IVF therapy to induce superovulation could negatively affect tissue regeneration of the endometrium by inhibiting the self-renewal, migration capacity, and pluripotency of endometrial stem cells in vivo.

## Discussion

Intensive studies on key regulatory factors and pathways that can affect the tissue repair capacity-related functions of endometrial stem cells may provide new insights into previously unexplained recurrent miscarriage or infertility related to endometrial factors. Among many cytokines and growth factors whose major functions in endometrial stem cells remain largely unknown, increasing attention has recently been devoted to the negative effects of FSH treatment due to its high usability in infertility treatments. During the superovulation process in most IVF strategies, FSH stimulates the secretion of estrogen, which in turn stimulates superovulation and subsequently leads to improved pregnancy rates. However, relatively high abortion rates and significantly low ongoing pregnancy rates (28.2% and 18.1%, respectively) [31] are among the most challenging points of the current recombinant FSH (rFSH)-based IVF protocol. At the level of the pituitary-ovarian axis, FSH can stimulate ovulation through estrogen secretion. Thus, it is presumed to subsequently increase pregnancy rates [14, 32]. However, for patients suffering from repeated implantation failure or recurrent pregnancy loss, another important factor to be considered for successful pregnancy is endometrial receptivity, which is presumed to be a critical beginning step for a successful embryo implantation process [33, 34]. Until recently, FSH-mediated effects on endometrial receptivity have historically been considered to have secondary (indirect) actions through FSH-induced estrogen. Interestingly, in addition to its previously known function in controlling the pituitary-gonadal axis, FSH itself and its receptors are also expressed in female extraovarian reproductive tissues such as the placenta [35] and the endometrium [27]. In this context, we hypothesized that these low ongoing pregnancy rates with FSH-based superovulation protocols could be related to the negative effects of consecutive FSH treatment on endometrial receptivity. Furthermore, new challenging questions have arisen regarding the possible direct effect of FSH on the repair capacity of tissue-resident stem cells, which play an essential role in local tissue regeneration and maintenance. Importantly, self-renewing local endometrial stem cells were not detected in nearly 42% of endometrial tissues with recurrent pregnancy loss compared to 11% of normal endometrial tissues [13]. In addition, although Chan et al. found no significant difference in the cloning efficiency between endometrial stem cells from endometriosis patients and normal women [36], the dysfunction of endometrial stem cells is likely to induce endometriosis by promoting angiogenesis and immunomodulation in response to various genetic or environmental factors [37, 38]. We thus investigated whether exogenous FSH treatment could directly inhibit tissue repair capacity-related functions of endometrial stem cells and consequently decrease endometrial receptivity. Currently, the potential effects of FSH on endometrial receptivity and successful pregnancy outcome remain controversial due to many different conflicting results. Some studies have observed positive effects of FSH [14, 39–41], whereas other results have shown negative effects of FSH [42–45]. In addition, although these studies have shown its direct effects in terminally differentiated endometrial cell models in vitro, the potential effects of FSH on the repair capacity-related functions of endometrial stem cells and its underlying molecular mechanisms remain unknown. To the best of our knowledge, this is the first study related to this issue. Previously, Pieri et al. found that FSH can promote the self-renewal and pluripotency of spermatogonial stem cells (SSCs) both in vitro and in vivo and that SSCs prestimulated with FSH have a better regenerative capacity to overcome infertility in a canine model [46]. Similarly, Patel et al. have shown that FSH treatment can significantly improve the self-renewal capacity and pluripotency-associated properties of SSCs [47]. Patel et al. also observed that FSH can increase clonal expansion and the expression of the stemness-related genes OCT4 and SOX2 in ovarian stem cells through FSH-R1 and FSH-R3 [48]. In contrast with these positive effects of FSH in other stem cell models, the results of the present study revealed for the first time that FSH treatment significantly inhibited various tissue repair capacity-related functions of endometrial stem cells, including their self-renewal, migration capacity, multilineage differentiation potential, and pluripotency, both in vitro (Fig. 1a–g) and in vivo (Fig. 7a–g). Self-renewal, migration capacity, multilineage differentiation potential, and pluripotency are essential functions for endometrial regeneration and maintenance [13, 49–52]. Unfortunately, the culture conditions or media compositions that can induce the differentiation of endometrial stem cells into various endometrial composing cells, such as myometrial and endometrial epithelial cells, are not currently established. Moreover, adipocyte and osteoblast differentiation of adult stem cells is a widely used and well-established analysis model to evaluate the differentiation capacity of certain types of adult stem cells [53]. Therefore, endometrial stem cells were differentiated into adipocytes and osteoblasts to investigate their multilineage differentiation capacity in vitro, not because their differentiation into adipocytes or osteoblasts itself is physiologically meaningful. We next investigated the molecular mechanism underlying these suppressive effects of FSH on endometrial stem cells. Previously, FSH was shown to inhibit prosurvival Akt and ERK1/2 signaling pathways involved in various beneficial functions of stem cells, such as growth (self-renewal) [54, 55], pluripotency/stemness [54, 56, 57], and migratory capacity [54, 58]. Indeed, the FSH-mediated suppressive effects on various tissue repair capacity-related functions of endometrial stem cells were significantly abolished by prestimulation with SC79, an Akt activator (Fig. 4a–e), or C6-ceramide, an ERK1/2 activator (Fig. 5a–e). These results suggest that the Akt and ERK1/2 signaling pathways might act as downstream mediators of the FSH-induced suppressive effects on various functions of endometrial stem cells.

Taken together, we found for the first time that FSH largely inhibited various regenerative potential-associated functions of endometrial stem cells, such as growth potential, migratory capacity, and pluripotency, both in vitro and in vivo. In addition, FSH inhibited the key prosurvival signaling pathways PI3K/Akt and FAK/ERK1/2. Blocking these signaling molecules with specific synthetic inhibitors markedly attenuated the FSH-induced inhibitory effects on various regenerative potential-associated functions of endometrial stem cells. The results of this study indicate that in addition to its previously known effects in ovarian follicles, FSH can directly inhibit the regenerative potential of endometrial stem cells through the Akt and ERK1/2 signaling pathways. Our findings may facilitate the development of more promising infertility treatment strategies by alleviating infertility drug-mediated inhibitory effects on various beneficial functions of endometrial stem cells and subsequent uterine receptivity.

## Supplementary information

Supplementary figures and legends
