# Supplementary material for: A novel role of follicle-stimulating hormone (FSH) in various regeneration-related functions of endometrial stem cells
Source: Exp Mol Med. 2022 Sep 18;54(9):1524–35. doi: 10.1038/s12276-022-00858-1 (PMC9534881; doi:10.1038/s12276-022-00858-1)
Supplement: Supplementary file 1 — Supplementary figures and legends [file 12276_2022_858_MOESM1_ESM.pdf]

# Supplementary figures and legends

## Supplementary figure 1

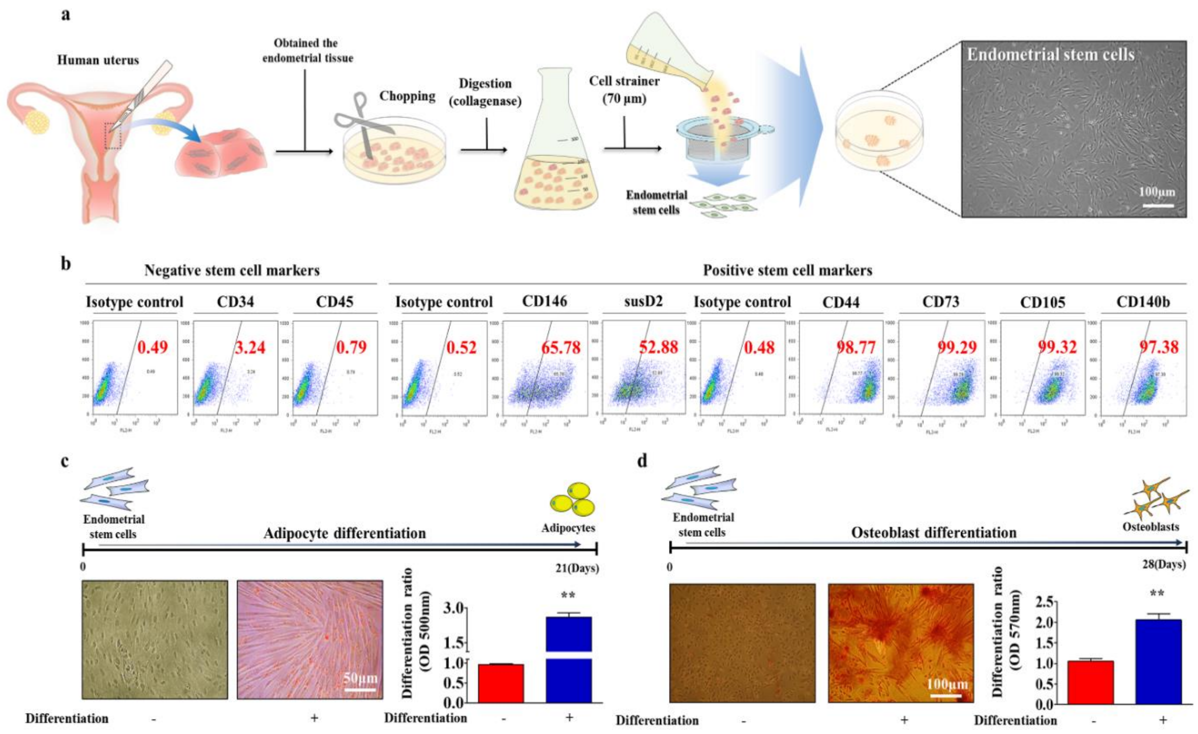


**Supplementary Fig. 1 Isolation and characterization of human endometrial stem cells from endometrial tissues.** Endometrial tissue was minced into small pieces, and then the small pieces were digested with type I collagenase. Isolated human endometrial stem cells were observed under an inverted phase-contrast microscope to assess their morphological characterization **(a)**. The isolated endometrial stem cells were analyzed using flow cytometry with various antibodies for identified stem cell markers (CD44, CD73, CD105, CD140b, CD146, and susD2) and several hematopoietic markers (CD34 and CD45) **(b)**. Their ability to differentiate into adipocytes **(c)** and osteoblasts **(d)** was analyzed using oil red O and alizarin red S staining, respectively. The relative quantification of calcium deposition and lipid droplet (LD) secretion from differencing cells were assessed by measuring the absorbance of the solubilized cells at 500 nm and 570 nm, respectively. All experiments were performed in triplicates, and the data has been presented as mean ± standard deviation (SD). *$p < 0.05$, **$p < 0.005$, and ***$p < 0.001$ (two-sample t-test).

**Supplementary figure 2**

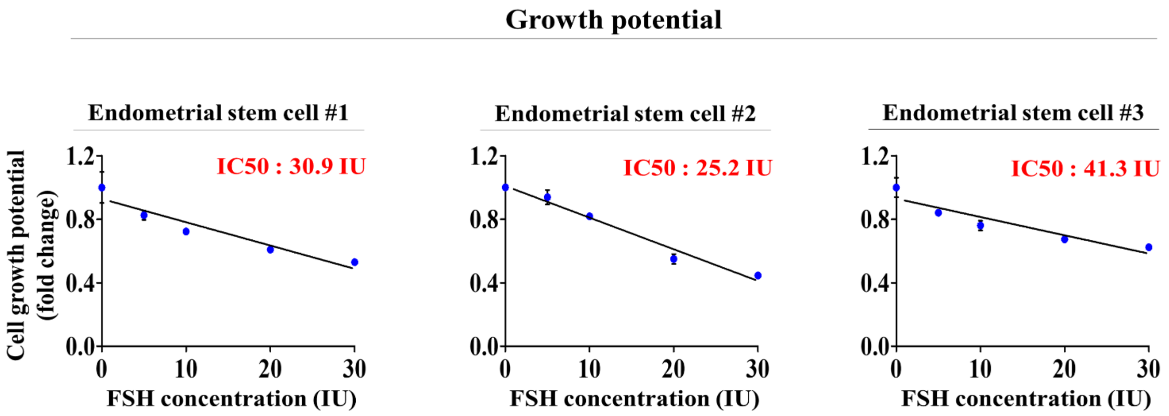


**Supplementary Fig. 2. $IC_{50}$, the concentration that inhibits 50% of endometrial stem cell proliferation.** Inhibition of cell viability by treatment with FSH for 72 hours was determined by a MTT assay in human endometrial stem cells. Cell viability (%) was calculated as a percent of the vehicle control.

**Supplementary figure 3**

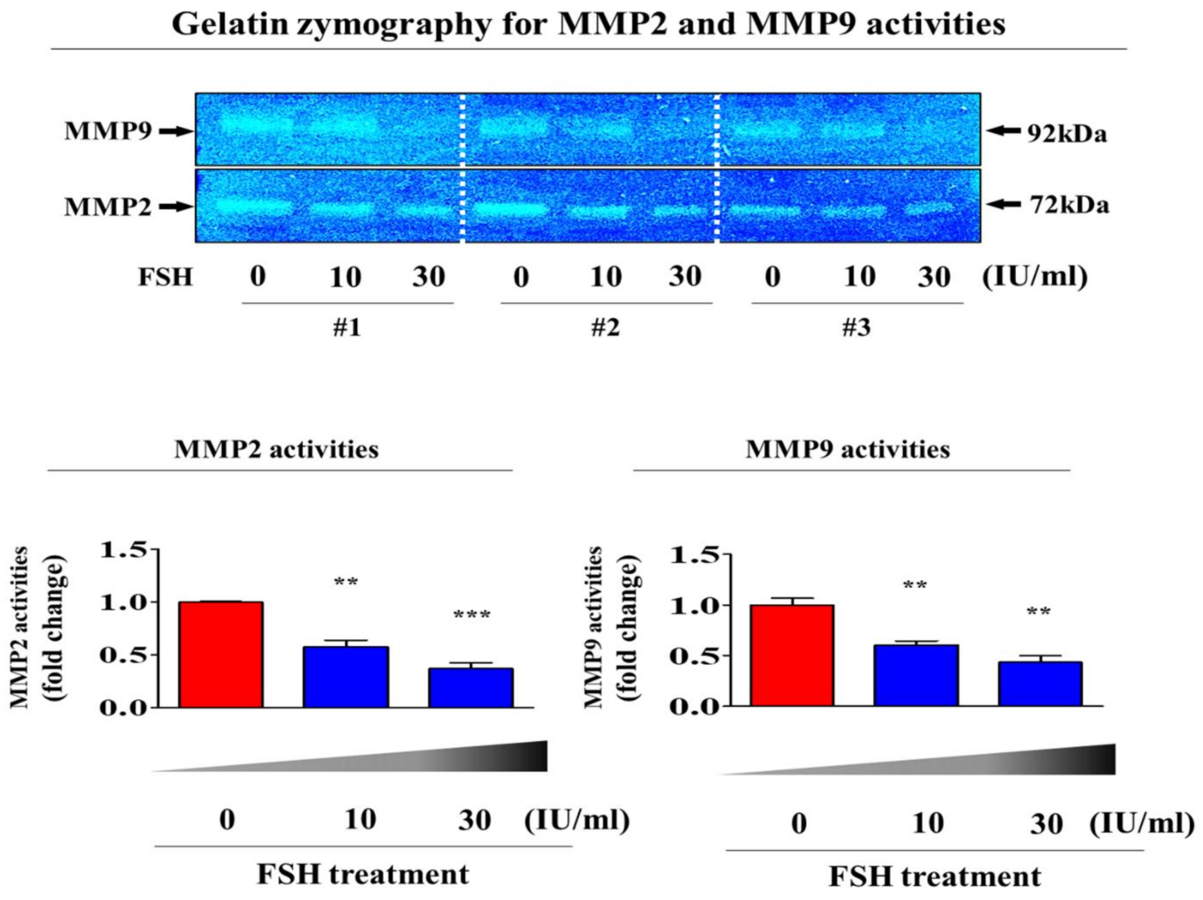


**Supplementary Fig. 3. FSH significantly decreases the activity of MMP-2 and MMP-9 in human endometrial stem cells.** Endometrial stem cells were treated with FSH (10 and 30IU) for 72 h, and then the effect of FSH on the activity of MMP-2 and MMP-9 was evaluated using gelatin zymography. Compared with those of the nontreated control group, the activities of MMP-2 and MMP-9 were significantly decreased by FSH treatment.

**Supplementary figure 4**

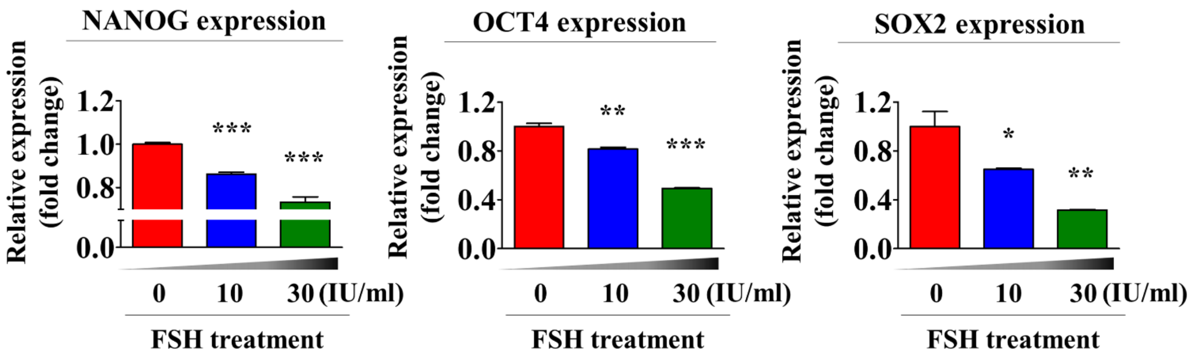


**Supplementary Fig. 4. Expression levels of various pluripotency-related genes in response FSH treatment.** Inhibitory effects of FSH treatment on mRNA levels of several pluripotency/stemness-related genes (NANOG, OCT4, and SOX2) were analyzed by real-time PCR. PPIA was used as a housekeeping gene for real-time PCR analysis. All experiments were performed in triplicates. Data are presented as mean ± standard deviation (SD). *, $p < 0.05$; **, $p < 0.005$; and ***, $p < 0.001$ (two-sample t-test).

## Supplementary figure 5

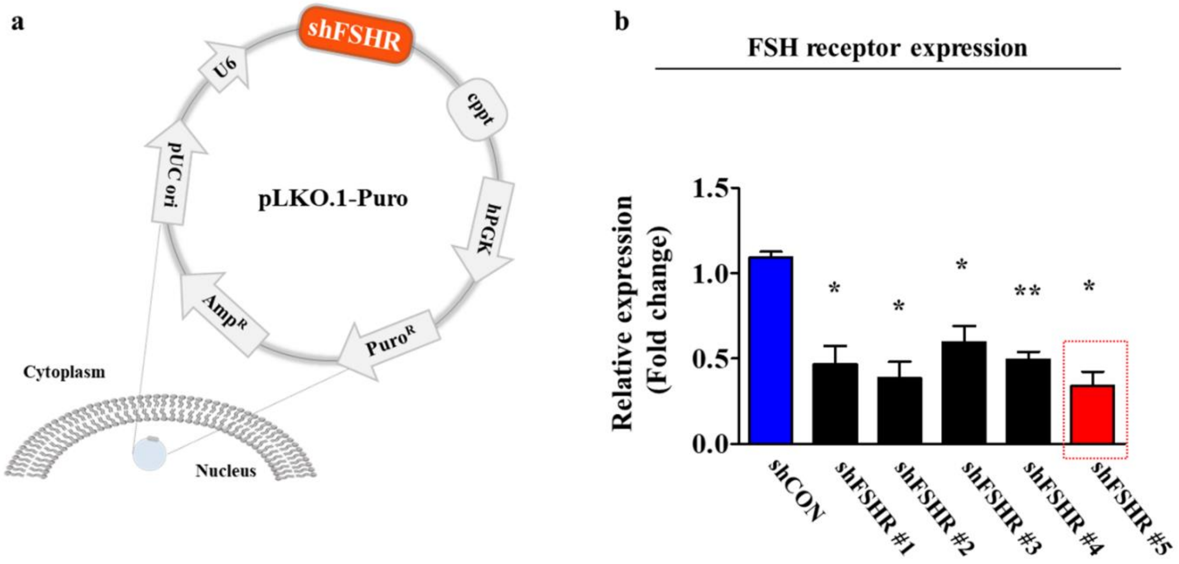


**Supplementary Fig. 5 Knockdown efficacy of several shRNA constructs specifically targeting FSHR.** Endometrial stem cells were transfected with several shRNA constructs #1, #2, #3, #4, or #5, which specifically target FSHR, or with a non-targeting shRNA control for non-specific effects **(a)**. FSHR shRNA construct #5, hereafter described as FSHR shRNA, was the most effective. The knockdown efficacy of FSHR was analyzed using real-time PCR at mRNA levels **(b).** HPRT was used as a reference gene to normalize gene expression. All experiments were performed in triplicates, and the data has been presented as mean ± standard deviation (SD). $*p < 0.05$, $**p < 0.005$, and $***p < 0.001$ (two-sample t-test).

**Supplementary figure 6**

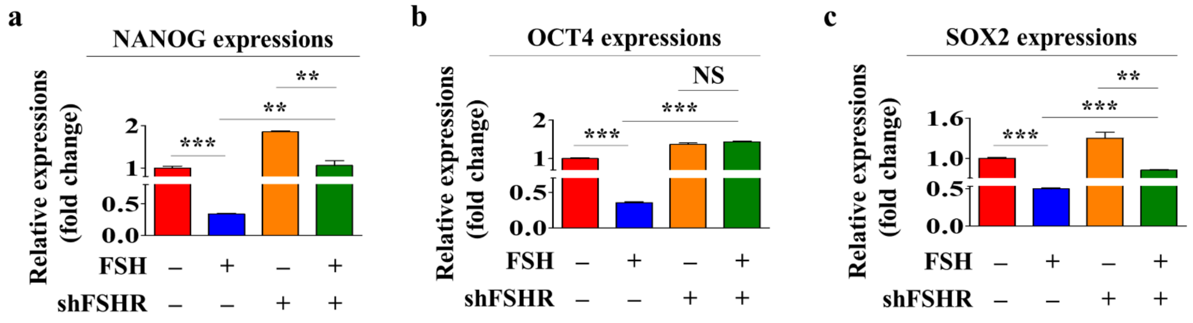


**Supplementary Fig. 6. FSH-mediated effects on expression levels of pluripotency-related genes were significantly decreased by FSHR depletion.** Abolishing effects of FSHR knockdown on mRNA levels of pluripotency/stemness-related genes NANOG, OCT4, and SOX2 were assessed by real-time PCR **(a-c)**. PPIA was used as a housekeeping gene for real-time PCR analysis. All experiments were performed in triplicates. Data are presented as mean ± standard deviation (SD). *, $p < 0.05$; **, $p < 0.005$; and ***, $p < 0.001$ (two-sample t-test).

## Supplementary figure 7

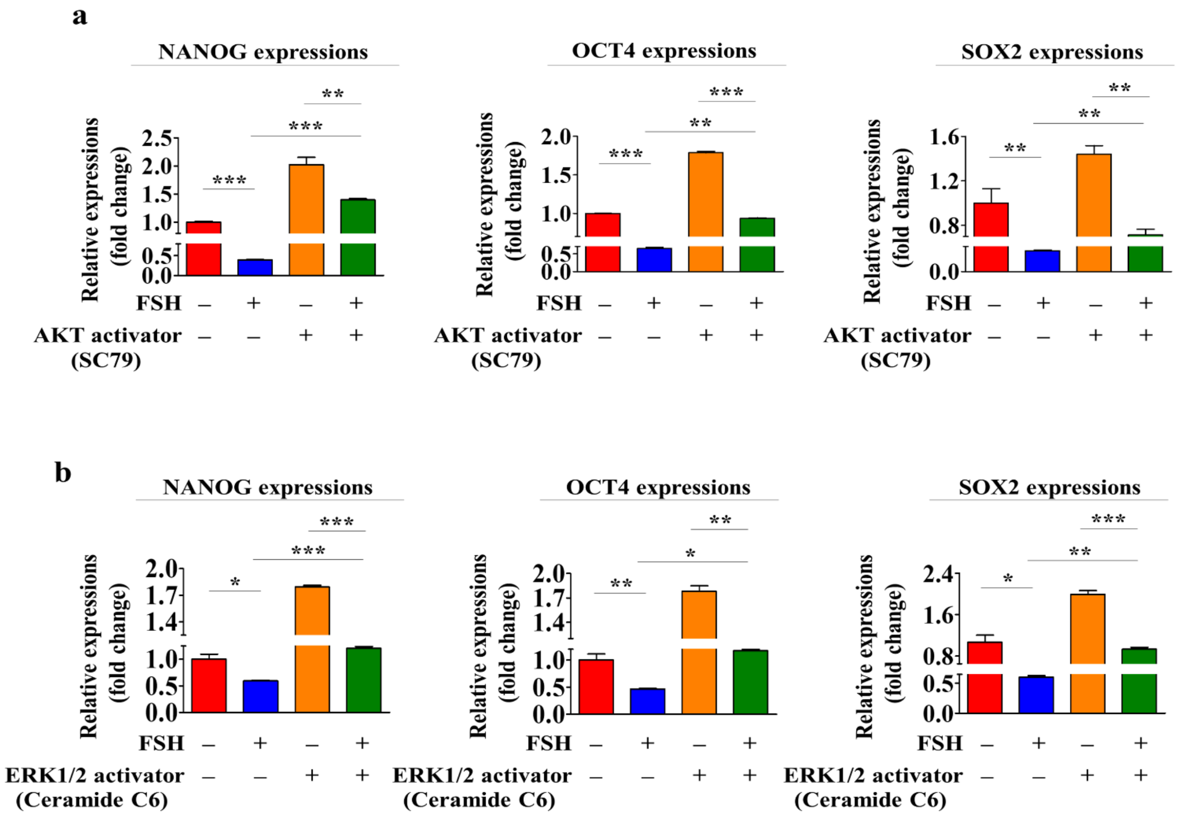


**Supplementary Fig. 7. The FSH-induced inhibitory effects on the expression of pluripotency-related genes were also significantly attenuated by SC79 or ceramide C6 pretreatment.** Abolishing effects of Akt activator SC79 (10 µM) **(a)** or ERK1/2 activator ceramide C6 (10 µM) **(b)** on FSH-induced changes in mRNA levels of pluripotency/stemness-related genes NANOG, OCT4, and SOX2 were analyzed by real-time PCR. PPIA was used as a housekeeping gene for real-time PCR analysis. All experiments were performed in triplicates. Data are presented as mean ± standard deviation (SD). *, $p < 0.05$; **, $p < 0.005$; and ***, $p < 0.001$ (two-sample t-test).

# Supplementary figure 8

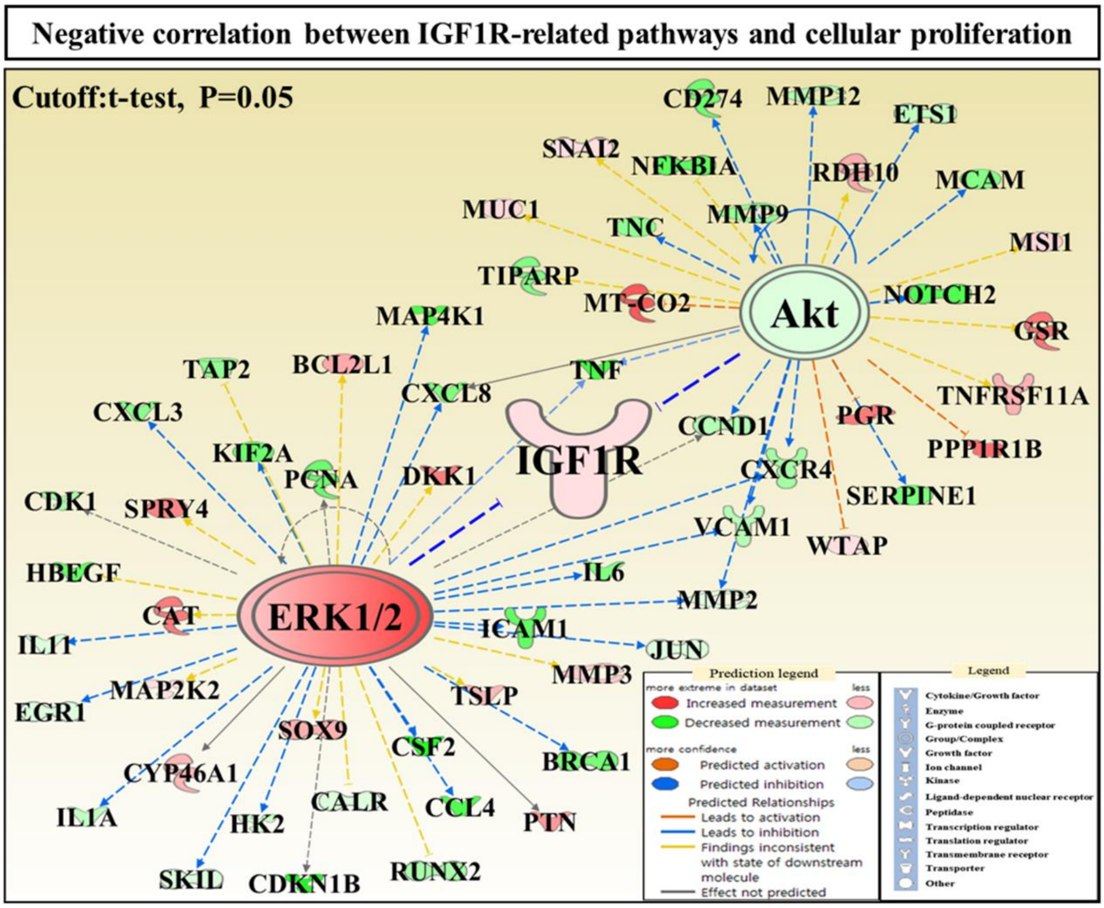


| Signaling pathways | Predicted activation group | # of Target molecules in dataset | Predicted activation state | Activation z-score | P-value of overlap |
|---|---|---|---|---|---|
| **Akt** | Non-proliferative cells (vs proliferative cells) | 37 | Inhibition | -1.937 | 9.41E-01 |
| **ERK1/2** | Non-proliferative cells (vs proliferative cells) | 57 | Inhibition | -2.145 | 9.10E-01 |

**Supplementary Fig. 8 The FSH-induced signaling networks of the IGF1R-related prominent proteins are positively correlated with self-renewal capacity.** The differential activation status (whether intermediates are inactivated or activated) of various signaling pathways, such as IGF1R (GSE63074) associated molecules/transcription factors, between proliferative cells and non-proliferative cells was analyzed using IPA software.

# Supplementary figure 9

**Positive correlation between PDGFA-related pathways and cellular proliferation**

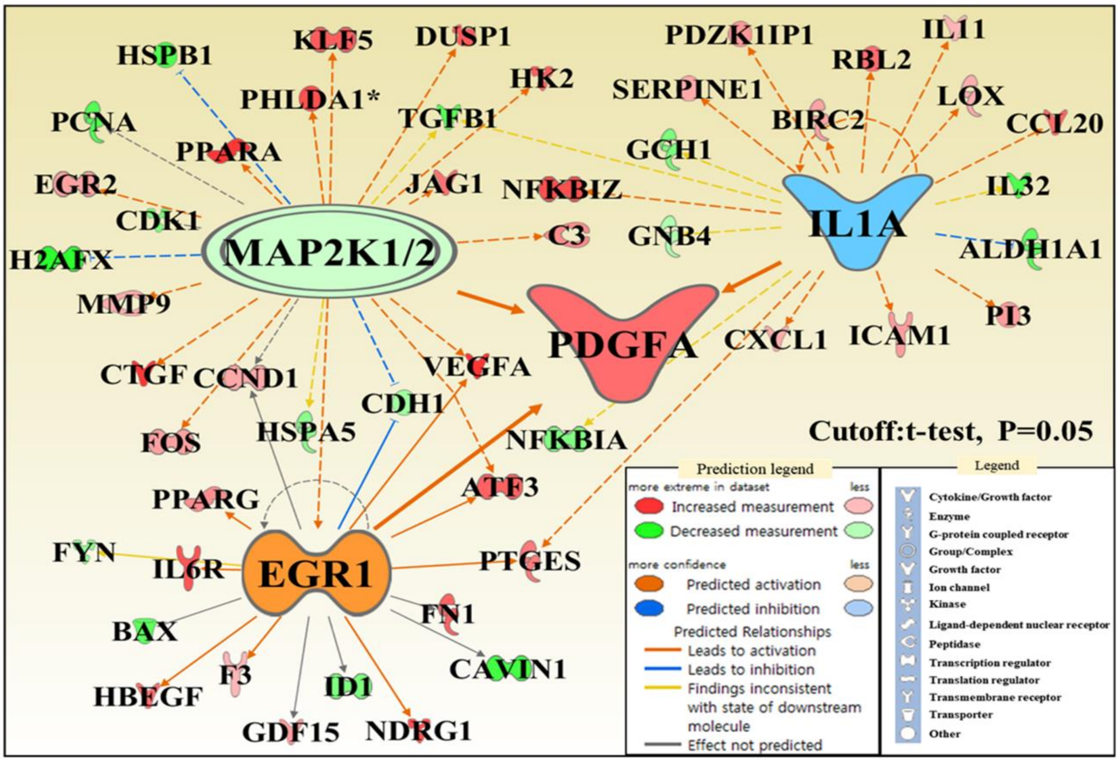


| Signaling pathways | Predicted activation group | # of Target molecules in dataset | Predicted activation state | Activation z-score | P-value of overlap |
|---|---|---|---|---|---|
| **MAP2K1/2** | Proliferative cells (vs non-proliferative cells) | 29 | Activation | 2.707 | 1.87E-01 |
| **EGR1** | Proliferative cells (vs non-proliferative cells) | 18 | Activation | 2.548 | 4.97E-01 |
| **IL1A** | Proliferative cells (vs non-proliferative cells) | 24 | Activation | 2.438 | 1.00E00 |

**Supplementary Fig. 9 The FSH-induced signaling networks of the PDGFA-related prominent proteins are positively correlated with self-renewal capacity.** The differential activation status (whether intermediates are inactivated or activated) of various signaling pathways, such as PDGFA (GSE28878) associated molecules/transcription factors, between proliferative cells and non-proliferative cells was analyzed using IPA software.

# Supplementary figure 10

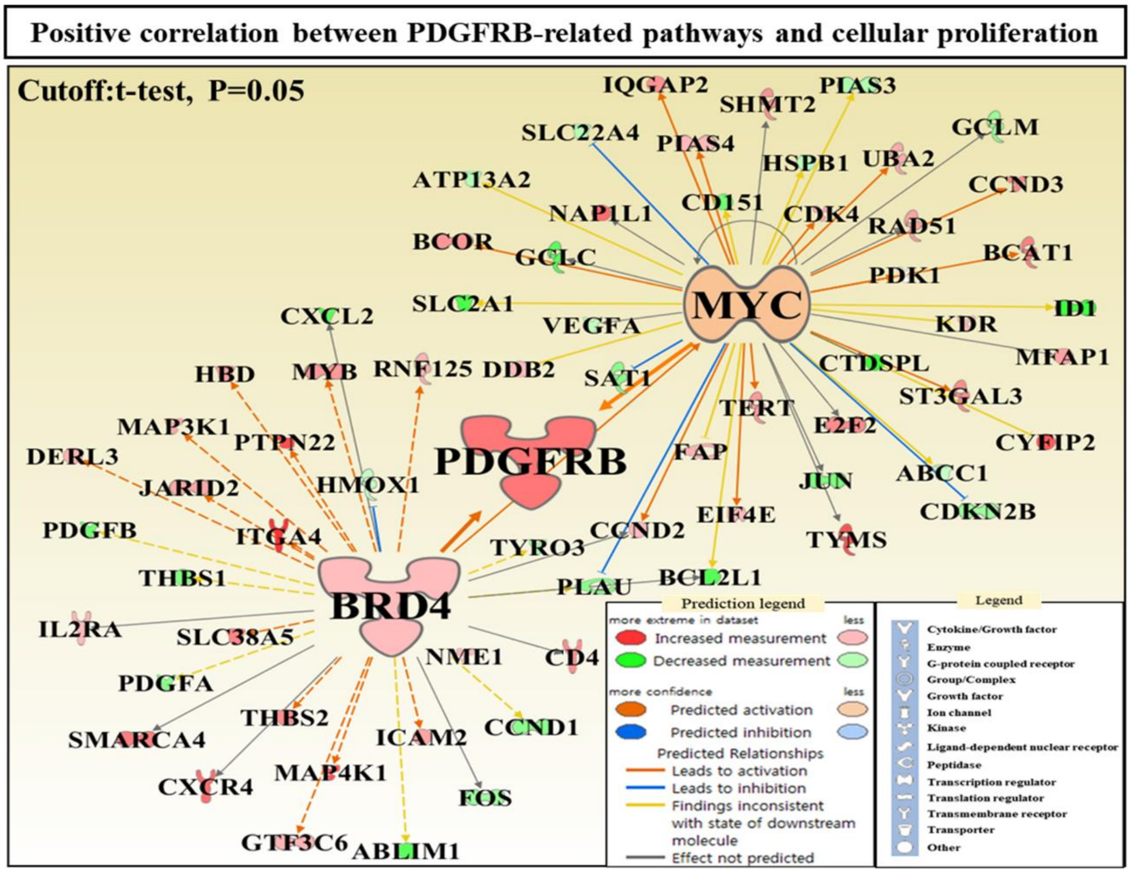


| Signaling pathways | Predicted activation group | # of Target molecules in dataset | Predicted activation state | Activation z-score | P-value of overlap |
|---|---|---|---|---|---|
| **BRD1** | Proliferative cells (vs non-proliferative cells) | 60 | Activation | 2.819 | 1.67E-01 |
| **MYC** | Proliferative cells (vs non-proliferative cells) | 110 | Activation | 2.347 | 3.12E-01 |

**Supplementary Fig. 10 The FSH-induced signaling networks of the PDGFRB-related prominent proteins are positively correlated with self-renewal capacity.** The differential activation status (whether intermediates are inactivated or activated) of various signaling pathways, such as PDGFRB (GSE36133) associated molecules/transcription factors, between proliferative cells and non-proliferative cells was analyzed using IPA software.

# Supplementary figure 11

**Negative correlation between EGF-related pathways and cellular proliferation**

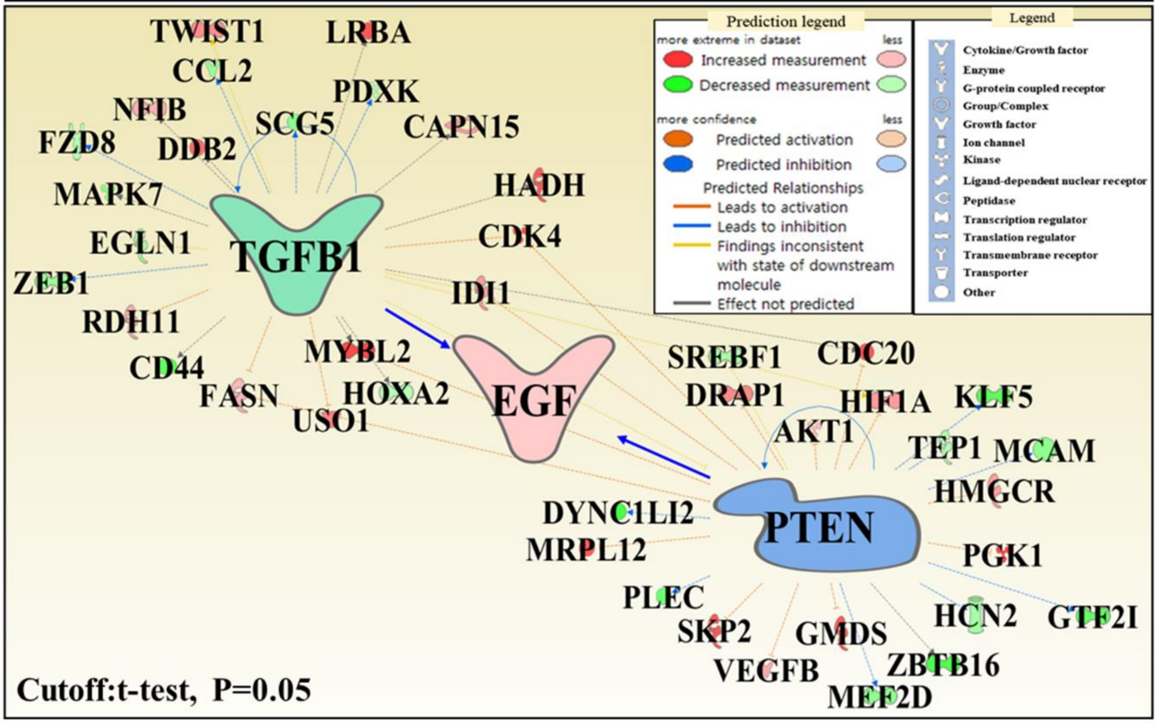


| Signaling pathways | Predicted activation group | # of Target molecules in dataset | Predicted activation state | Activation z-score | P-value of overlap |
|---|---|---|---|---|---|
| **PTEN** | Non-proliferative cells (vs proliferative cells) | 127 | Inhibition | -5.059 | 9.84E-01 |
| **TGFB1** | Non-proliferative cells (vs proliferative cells) | 475 | Inhibition | -3.103 | 9.39E-01 |

**Supplementary Fig. 11 The FSH-induced signaling networks of the EGF-related prominent proteins are positively correlated with self-renewal capacity.** The differential activation status (whether intermediates are inactivated or activated) of various signaling pathways, such as EGF (GSE62564) associated molecules/transcription factors, between proliferative cells and non-proliferative cells was analyzed using IPA software.

# Supplementary figure 12

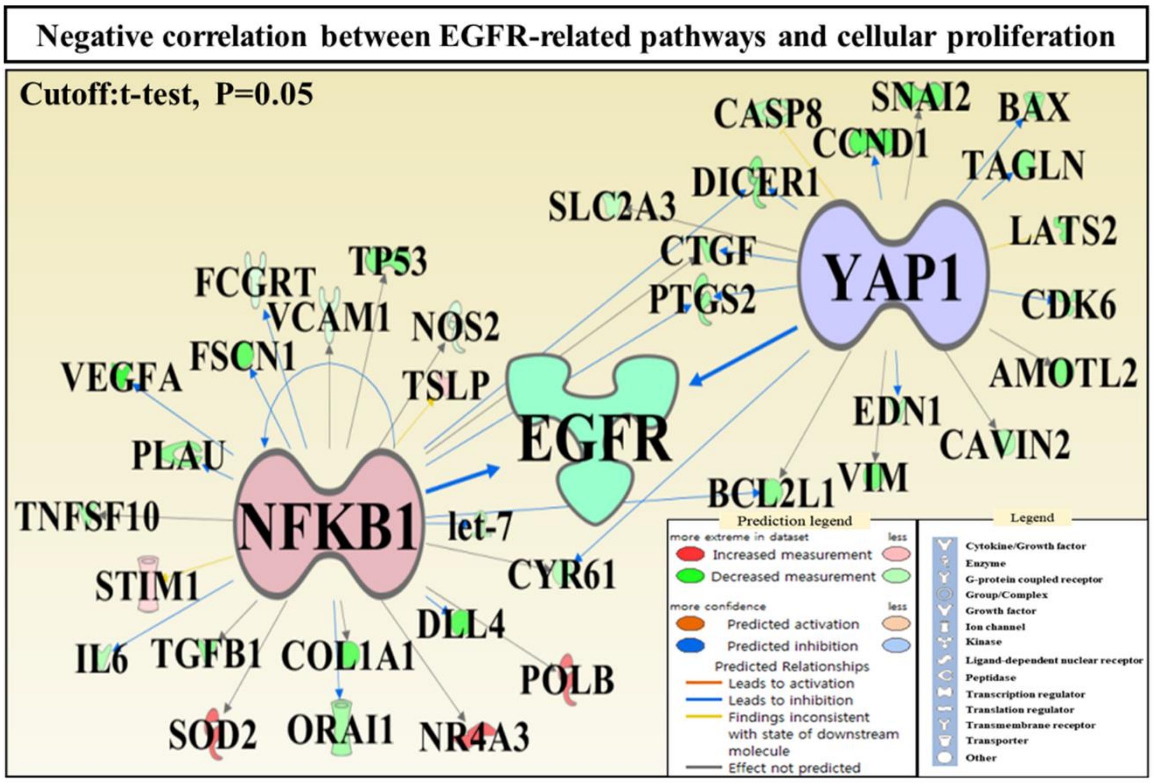


| Signaling pathways | Predicted activation group | # of Target molecules in dataset | Predicted activation state | Activation z-score | P-value of overlap |
|---|---|---|---|---|---|
| **YAP1** | Non-proliferative cells (vs proliferative cells) | 18 | Inhibition | -2.226 | 1.00E00 |
| **NFKB1** | Non-proliferative cells (vs proliferative cells) | 25 | Inhibition | -2.588 | 1.00E00 |

**Supplementary Fig. 12 The FSH-induced signaling networks of the EGFR-related prominent proteins are positively correlated with self-renewal capacity.** The differential activation status (whether intermediates are inactivated or activated) of various signaling pathways, such as EGFR (GSE62564) associated molecules/transcription factors, between proliferative cells and non-proliferative cells was analyzed using IPA software.

# Supplementary figure 13

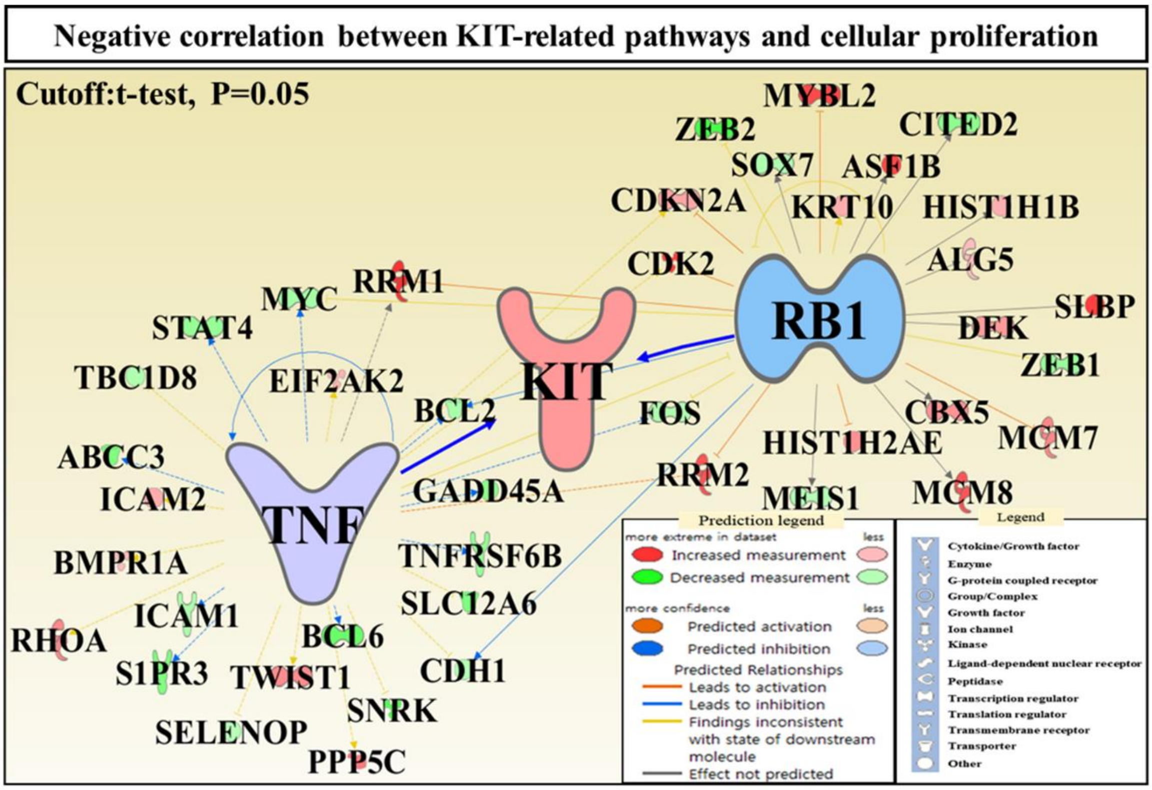


| Signaling pathways | Predicted activation group | # of Target molecules in dataset | Predicted activation state | Activation z-score | P-value of overlap |
|---|---|---|---|---|---|
| **TNF** | Non-proliferative cells (vs proliferative cells) | 383 | Inhibition | -3.909 | 9.51E-01 |
| **RB1** | Non-proliferative cells (vs proliferative cells) | 109 | Inhibition | -4.267 | 9.86E-01 |

**Supplementary Fig. 13 The FSH-induced signaling networks of the KIT-related prominent proteins are positively correlated with self-renewal capacity.** The differential activation status (whether intermediates are inactivated or activated) of various signaling pathways, such as KIT (GSE62564) associated molecules/transcription factors, between proliferative cells and non-proliferative cells was analyzed using IPA software.

Supplementary figure 14

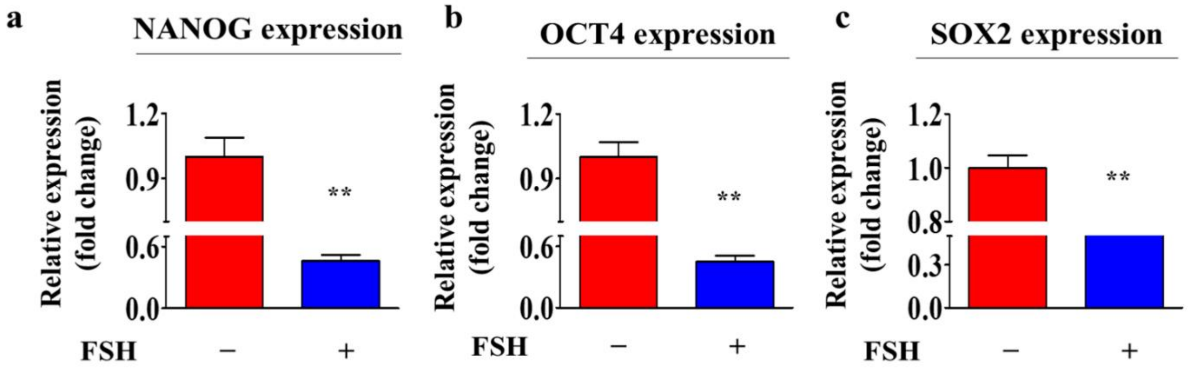


**Supplementary Fig. 14 Expression levels of various pluripotency-related genes in response FSH exposure *in vivo*.** FSH-induced suppression of several pluripotency-related genes (NANOG, OCT4, and SOX2) *in vivo* was analyzed using real-time PCR **(a-c)**. PPIA was used as a housekeeping gene for real-time PCR analysis. All experiments were performed in triplicates. Data are presented as mean ± standard deviation (SD). *, $p < 0.05$; **, $p < 0.005$; and ***, $p < 0.001$ (two-sample t-test).

## Supplementary figure 15

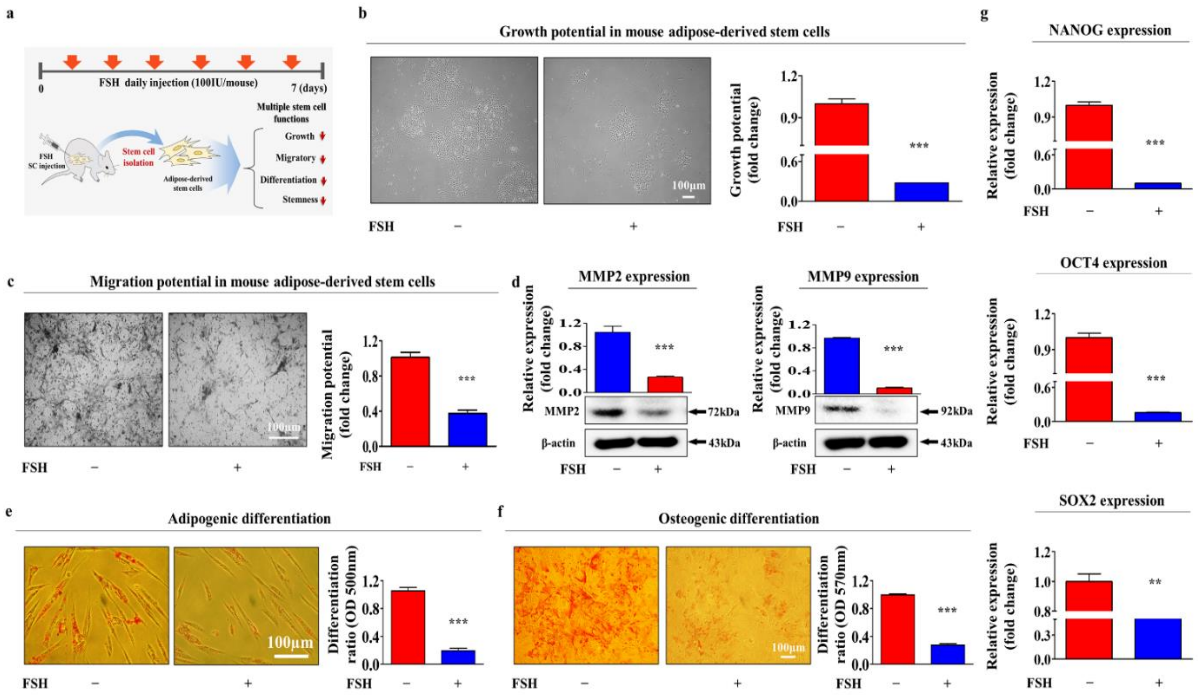


**Supplementary Fig. 15 FSH treatment markedly suppresses various tissue repair capacity of adipose tissue-derived stem cells *in vivo*.** A schematic diagram of overall experimental protocols as described in the section of 'Materials and Methods' is presented **(a)**. The mice were intravenously treated with FSH (100IU/mouse daily for 7 consecutive days), and then tissue resident stem cells were isolated from mouse adipose tissues using our collagenase-based primary culture method. After isolation of mouse adipose tissues-derived stem cells, they were cultured *in vitro* either under continuous FSH (30 IU/ml) treatment or non-FSH treatment conditions to properly mimic the *in vivo* environment of FSH exposure. The subsequent inhibition of cell proliferation was assessed by MTT assays **(b)**. The FSH-mediated suppression of migration capacity *in vivo* was then measured using Transwell assays **(c)** and western blotting for MMP-2 and -9 **(d)**. The FSH-mediated suppression of adipocyte **(e)** and osteoblast **(f)** differentiation *in vivo* were assessed by oil red O and alizarin red S staining, respectively. The relative quantification of calcium deposition and lipid droplet (LD) secretion from differencing cells were assessed by measuring the absorbance of the solubilized cells at 500 nm and 570 nm, respectively. The FSH-induced suppression of several pluripotency-related genes (NANOG, OCT4, and SOX2) *in vivo* was analyzed using real-time PCR **(g)**. β-actin was used as an internal control to

normalize protein expression. HPRT was used as a reference gene to normalize gene expression. All experiments were performed in triplicates, and the data has been presented as mean ± standard deviation (SD). *$p < 0.05$, **$p < 0.005$, and ***$p < 0.001$ (two-sample t-test).
